# Synthetic Oleanane Triterpenoids Reduce Tumor Growth and Promote an Anti-Tumor Immune Response Independent of Cancer *KEAP1* Mutational Status

**DOI:** 10.3390/antiox14121406

**Published:** 2025-11-26

**Authors:** Christopher J. Occhiuto, Jessica A. Moerland, Karen T. Liby, Ana S. Leal

**Affiliations:** 1 Department of Pharmacology and Toxicology, Michigan State University, East Lansing, MI 48824, USA; occhiut1@msu.edu; 2 Department of Pediatrics, Indiana University School of Medicine, Indianapolis, IN 46202, USA; jamoerla@iu.edu; 3 Division of Hematology and Oncology, Indiana University School of Medicine, Indianapolis, IN 46202, USA; ktliby@iu.edu

**Keywords:** lung cancer, KEAP1, NRF2, CDDO-Me, triterpenoids, immune system, macrophages

## Abstract

KEAP1 negatively regulates the cytoprotective factor NRF2 and is commonly inactivated in lung cancer cells. Loss-of-function *KEAP1* mutations in cancer cells contribute to NRF2 activation and tumor immune evasion through immunosuppression and drug resistance. Counterintuitively, treatment with synthetic oleanane triterpenoids, potent NRF2 activators, reduces the pre-clinical tumor burden. This suggests the functional target of these drugs in cancer models is not the cancer cells but another tumor immune microenvironment (TIME) cell population. The anti-tumor potential of cells within the TIME, particularly macrophages, is potentiated by triterpenoid treatment in cancers with wild-type *KEAP1* status. As *KEAP1*-mutant cancers show reduced tumor immune responses, triterpenoid-mediated immune stimulation may particularly benefit these cases, but this has not been investigated. To characterize the immunomodulatory effects of triterpenoids in *KEAP1*-mutant lung cancer, we studied tumor-educated bone marrow-derived macrophages (TE-BMDMs) and lung cancer models treated with the triterpenoids CDDO-Me or omaveloxolone. RNA-sequencing of TE-BMDMs cultured in *KEAP1* KO compared to WT cancer-conditioned media had enhanced tumor-promoting phenotypes, which reversed with CDDO-Me treatment. Similarly, subcutaneous *KEAP1* KO tumors were larger and more immune-suppressed compared to WT tumors. Both CDDO-Me and omaveloxolone reduced the tumor burden and improved immune cell phenotypes within the TIME independent of *KEAP1* mutational status.

## 1. Introduction

Lung cancer poses a significant burden on patients’ lives and is frequently associated with poorer outcomes compared with other types of cancer. In 2025, an estimated 226,650 new cases of lung and bronchus cancers and 60,540 deaths are projected to occur. The average 5-year survival rate for all stages is below 30%, with advanced disease diagnosis at 9% [1]. While disease staging is a strong prognostic indicator, further insight can be gained from genomic mutations in driver genes that are associated with treatment resistance or those that offer survival benefits to cancer cells [2,3]. These mutations can lead to differential regulation of the tumor immune microenvironment (TIME), whose composition and cellular phenotypes are critical in shaping disease progression and response to therapies [4,5,6]. Immune-modifying cancer therapies have grown in prominence and are now part of the first-line regimen for several cancers, including lung cancer [7]. While immunotherapy has been very effective in a subset of patients, refractory patient populations remain, warranting further investigation into new treatment modalities and mechanisms of therapeutic resistance.

Kelch-like ECH-associated protein 1 (KEAP1) is mutated in 17% of lung adenocarcinomas [8] and confers resistance to platinum therapy and immunotherapies [9,10,11]. KEAP1 is the negative regulator of the cytoprotective nuclear factor erythroid 2-related factor 2 (NRF2) pathway [12]. While the activation of the NRF2 transcriptional pathway protects normal cells from malignant transformation, cancer cells can use this signaling axis to promote survival, drug resistance, and immune evasion. Lung cancer patients with *KEAP1* mutations present with more aggressive disease and have decreased survival rates [13,14], likely due to constitutive NRF2 pathway activation. Cancer cell-specific NRF2 pathway-activating mutations are correlated with immunosuppressive TIMEs, generating low treatment responses [15]. The literature on *KEAP1*-mutant cancers is growing [16,17,18,19,20], but more knowledge is needed to ascertain which treatments would provide the most benefit in this subset of lung cancer.

Synthetic oleanane triterpenoids are derived from natural products and are potent activators of NRF2 in cells [21]. Triterpenoid derivatives have demonstrated significant chemopreventive and treatment efficacy in pre-clinical cancer models [22,23,24,25,26,27,28], although the clinical development for cancer has been limited. Prominent members of this class include CDDO-Me (bardoxolone methyl) [29] and the recently FDA-approved omaveloxolone [30]. While CDDO-Me has been more thoroughly characterized in the cancer context, its effectiveness is thought to function by stimulating anti-cancer immune responses [31,32,33].

The KEAP1/NRF2 axis, by virtue of its antioxidant and cytoprotective effects, is a double-edged sword in cancer treatment. Its activation leads to cell type-specific consequences: activation of the NRF2 pathway provides resistance to genomic damage in normal cells but gives a survival advantage to mutant cancer cells [34,35,36,37]. Moreover, NRF2 activation in the immune microenvironment drives strong anti-tumor immunity [38], potentially through enabling their tolerance to reactive oxygen species (ROS)-abundant environments. The difference in cellular responses complicates the prediction of outcomes when treating a *KEAP1*-mutant cancer with an NRF2-activator like CDDO-Me, since the known NRF2 effects on cancer and immune cell biology are opposing. Additionally, the nuances separating constitutive genetic NRF2 activation from transient pharmacological NRF2 activation demand the careful evaluation of this potential clinical scenario. The immunosuppressive microenvironment in *KEAP1*-mutant lung adenocarcinoma presents a promising target for triterpenoid derivatives to enhance anti-cancer immune functions. However, this treatment paradigm has not been explored. We posit that the anti-cancer benefit derived from systemic triterpenoid treatment targeting TIME cells will outweigh the cancer cell survival advantage since the cancer cells will likely have already achieved maximal NRF2 pathway activation through genetic *KEAP1* mutation. This manuscript investigates how synthetic triterpenoid derivatives affect *KEAP1*-mutant lung cancer growth, with a specific focus on the role of the immune system.

In murine models of lung carcinogenesis, we previously recognized that macrophages are associated with the immunosuppressive phenotype of *KEAP1*-mutant lung cancer [39]. Furthermore, we found that CDDO-Me repolarizes macrophages to an anti-cancer phenotype [27,33]. Our current study builds on these prior findings and directly evaluates the efficacy of triterpenoid derivatives in treating *KEAP1*-mutant murine lung cancer. Specifically, our objectives were to investigate how triterpenoids modulate macrophage phenotypes and to evaluate triterpenoid efficacy in reducing the tumor burden in *KEAP1*-mutant mouse models. We report that *KEAP1*-mutant LL2 murine lung cancer cells directly regulate macrophages to promote their pro-tumor differentiation and the abundance of this macrophage subset within the tumor microenvironment. However, this effect was reversed by treatment with CDDO-Me and correlated with decreased tumor burden. Further, the clinically approved NRF2 activator omaveloxolone exhibited a similar treatment efficacy and restructuring of immune phenotypes. Therefore, this study allays concerns about pro-cancer survival benefit by demonstrating the efficacy of triterpenoid derivatives in a murine model of *KEAP1*-mutant lung cancer through reshaping the tumor immune microenvironment.

## 2. Materials and Methods

### 2.1. Cell Lines and Tissue Culture

LL2 (Lewis lung carcinoma, CRL-1642-LUC2) cells were purchased from the American Type Culture Collection (ATCC; Manassas, VA, USA). Cells were cultured in Dulbecco’s Modified Eagle Medium (DMEM, Corning; Steuben County, NY, USA) supplemented with 10% fetal bovine serum (FBS, VWR; Radnor, PA, USA) and 1% penicillin and streptomycin (P/S, Corning, Steuben County, NY, USA). LL2 WT, *KEAP1* KO, and *NRF2* KO lines were maintained in a humidified incubator with 5% atmospheric CO_2_ at 37 °C and used within five to six passages after thawing. Mutations were introduced using nucleofection of CRISPR plasmids co-expressing the Cas9 enzyme and guide RNAs as described previously [39].

### 2.2. Isolation of Bone Marrow-Derived Macrophages

C57BL/6 mice were sacrificed through CO_2_ asphyxiation. Femurs and tibias were harvested under sterile conditions and bone marrow was flushed out using DMEM with 10% FBS and 20 ng/mL recombinant M-CSF (BioLegend; San Diego, CA, USA). The suspension of progenitor cells was split evenly and approximately 500 K cells/well plated into six-well plates in 2 mL media. Plates were then incubated at 5% CO_2_ at 37 °C for 5 days, washed 3× in PBS, and fresh 2 mL DMEM with 10% FBS and 20 ng/mL M-CSF added. After an additional 48 h, naïve macrophages were ready for experimental use.

### 2.3. Conditioned Media Treatment of Macrophages

Confluent 100 mm dishes of LL2 lung cancer cells (WT, *KEAP1* KO, and *NRF2* KO) were split 1:4 in 15 mL DMEM with 10% FBS. An additional plate of media without cells was included as a control for naïve macrophages. After 48 h, the conditioned media (CM) was harvested and cleared through centrifugation at 500× *g* for 5 min. Cleared cancer CM was added to naïve macrophages totaling 75% CM and 25% fresh DMEM. A total of 100 nM CDDO-Me or vehicle control was added to macrophages and incubated for 24 h. RNA was harvested at the endpoint for three independent samples in each treatment group.

### 2.4. RNA Processing and mRNA Sequencing Analysis

Total RNA was extracted from macrophages using RNeasy Mini Spin Columns (QIAGEN Inc.; Hilden, Germany) according to manufacturer’s protocol. Sample purity was analyzed on a Nanodrop 2000 (ThermoFisher; Waltham, MA, USA). Samples with purity ratios 260/280 > 2.0 and 260/230 > 1.8 were submitted for sequencing. RNA was shipped to Novogene (Sacramento, CA, USA) for RNA QC, mRNA poly-A capture, library preparation, sequencing, and read filtering. Sequencing parameters included 150 bp paired-end reads and an average depth of 50 M. Files were received in .fastq format pre-filtered for adapter-related and low-quality reads. Read pseudo-alignment was completed using Kallisto [40,41] v0.51.0 to the Mus musculus GRCm39 cDNA genome. The dataset was filtered for low read counts, TMM normalized, and differential gene expression was calculated (limma [42,43] v3.58.1). Pathway analysis used clusterProfiler [44,45] v4.10.1 and QIAGEN IPA software (Fall Release: 29 September 2024, QIAGEN Inc., https://digitalinsights.qiagen.com/IPA, accessed on 1 October 2024).

### 2.5. RT-qPCR

RNA was isolated as above and the High-Capacity Reverse Transcription cDNA Kit (Applied Biosystems; Waltham, MA, USA) was used to convert 500 ng RNA to cDNA according to manufacturer’s protocol. SYBR Green (Applied Biosystems) Master Mix and indicated primer sets were used for amplification on a QuantStudio 6 Pro Real Time PCR System (Applied Biosystems). Primers were purchased from IDT (Coralville, IA, USA): *Gapdh* (F: CATCACTGCCACCCAGAAGACTG, R: ATGCCAGTGAGCTTCCCGTTCAG), *Arg1* (F: GTGAAGAACCCACGGTCTGT, R: AGAAAGGACACAGGTTGCCC), *Il6* (F: CACGGCCTTCCCTACTTCAC, R: TGCAAGTGCATCATCGGTGT) or QIAGEN: *Cxcl5* (PPM02966F-200). Relative gene expression was quantified using ^ΔΔCT^, normalized to *Gapdh* internal controls and the media-only treated macrophage group.

### 2.6. Mice and Flank Tumor Model

All experiments using mice were approved under Indiana University Institutional Animal Care and Use Committees (IACUC), protocol number 23143. In total, 5 × 10^5^ cells of each LL2 lung cancer line were suspended in sterile saline and injected into the right flank of 8-week-old, female C57BL/6 mice. When the tumors reached a palpable size (approximately 1 × 1 mm), the mice were fed a diet containing vehicle (ethanol) or 80 mg/kg of diet CDDO-Me or omaveloxolone (equivalent to 20 mg/kg body weight). Treatment diets were made by dissolving CDDO-Me (or omaveloxolone) in 12.5 mL ethanol and mixed with 37.5 mL Neobee oil (Spectrum Chemical; New Brunswick, NJ, USA). This combination was added to one kg 5002 powdered rodent meal (LabDiet; Gray Summit, MO, USA) and mixed using a KitchenAid mixer. The mice on the triterpenoid treatment received the diet intermittently: 4-day treatment, 3-day vehicle diet break, then return to treatment diet for the remainder of study. The tumors were measured daily using calipers and body weights were assessed twice per week to monitor tumor growth and toxicity, respectively. Mice were euthanized 10 days from treatment initiation. The study was conducted on a rolling enrollment basis.

Ten mice were used per group, except for the omaveloxolone treatment group where n = 5, resulting in a total of 65 mice. The sample size per group was determined using a power calculation based on pilot data from our laboratory. Tumor volumes for WT, *KEAP1* KO, and *NRF2* KO groups (means: 359, 769, and 180 mm^3^; standard deviation: 340) were used to compute the required sample size for a one-way ANOVA with α = 0.05 and power = 0.85 via an online power calculator. Mice were randomized for both cancer cell inoculation and treatment assignment. All mice were included in the analysis; no inclusion or exclusion criteria were applied. Potential confounders (e.g., cage location and order of tumor measurements) were not controlled. The study was open label. Mice were maintained under specific pathogen-free conditions with a 12 h light–dark cycle and were provided with food and water ad libitum. All efforts were made to minimize animal suffering. Mice were euthanized by CO_2_ asphyxiation at the study endpoint or when humane endpoints defined by the IACUC were reached, including when tumor ulceration occurred or tumor volume exceeded 2000 mm^3^. Mice were monitored for these findings daily.

### 2.7. Immunophenotyping

Tumors were harvested and immediately prepared for flow cytometry. Tissues were minced using sterile scissors and digested in DMEM containing collagenase (300 U/mL, Sigma-Aldrich; St. Louis, MO, USA) and DNAse (2 U/mL, Calbiochem; San Diego, CA, USA) with a gentleMACS Octo Dissociator with Heaters (Miltenyi Biotec; Gaithersburg, MD, USA). The pre-installed 37C_m_TDK_2 program was run for dissociation. Cell suspensions were cleared of debris using 40 μm cell strainers and red blood cells lysed in RBC lysis buffer (150 mM NH_4_Cl, 14.1 mM NaHCO_3_, 0.1 mM EDTA, pH 7.3, Sigma-Aldrich). Between the subsequent steps, cells were washed in phosphate-buffered saline containing 0.1% (*w*/*v*) bovine serum albumin (Sigma-Aldrich; St. Louis, MO, USA). Cells were stained for 20 min with Live/Dead Near-IR Viability dye (Invitrogen; Waltham, MA, USA) and Fc receptors were blocked in TruStain FcX anti-mouse CD16/CD32 (BioLegend; San Diego, CA, USA) for 10 min. Cells were stained for 30 min in a 1:1 solution of Brilliant Violet buffer (BD Bioscience; Franklin Lakes, NJ, USA): PBA (phosphate-buffered saline, 0.5% BSA, and 0.1% sodium azide) using an antibody cocktail consisting of the following: αCD45 (BV 510; #103138), αCD24 (BV 605; #101827), αCD64 (BV 711; #139311), αCD11b (PE-Cy7; #101216), αCD11c (PE-Cy5; #117316), αCD206 (BV 421; #141717), αCD25 (PE; #101904), αCD107a (Alexa Fluor 647; #121610), αPD-L1 (PE-Dazzle 594; #124324), αI-A/I-E (BV 650; #107641), αCD8 (PerCP-Cy5.5; #100733), αCD4 (FITC; #100405), αLy6C (APC; # 128015), αLy6G (Pacific Blue; # 127611), and αCD69 (BV 785; # 104543). All antibodies were purchased from BioLegend and used at the manufacturer’s recommended dilution. Cells were permeabilized using the Cytofix/Cytoperm Fixation/Permeabilization kit (BD Biosciences) for intracellular αFoxP3 (Alexa Fluor 700; #56-5773-80, Invitrogen) staining. Data were acquired on a four-laser Cytek Aurora spectral flow cytometer (Violet 405 nm, Blue 488 nm, Yellow-Green 561 nm, and Red 640 nm) and analyzed using FlowJo v10.9.0 with a published and optimized gating strategy [46].

### 2.8. Statistical Analysis

R Studio [47] 2024.04.0 + 735 “Chocolate Cosmos” running R v4.3.2 was used for sequencing analysis. The sequencing data were statistically analyzed using the relevant statistical models that are pre-built into each software package with standard settings. Differentially expressed genes were exported to Qiagen IPA software for further statistical analyses. For the in vivo experiments, mice were randomized to experimental groups on a rolling enrollment basis. Non-sequencing data was analyzed in GraphPad v10.4.2. The statistical test used for analysis is indicated in the figure legend. Assumptions for statistical tests were validated within GraphPad. Data are graphed as mean +/− SE unless otherwise indicated. *p* < 0.05 was deemed significant.

## 3. Results

### 3.1. KEAP1 KO Lung Cancer Cells Direct Pro-Tumor Polarization of Bone Marrow-Derived Macrophages In Vitro

**Figure 1:**
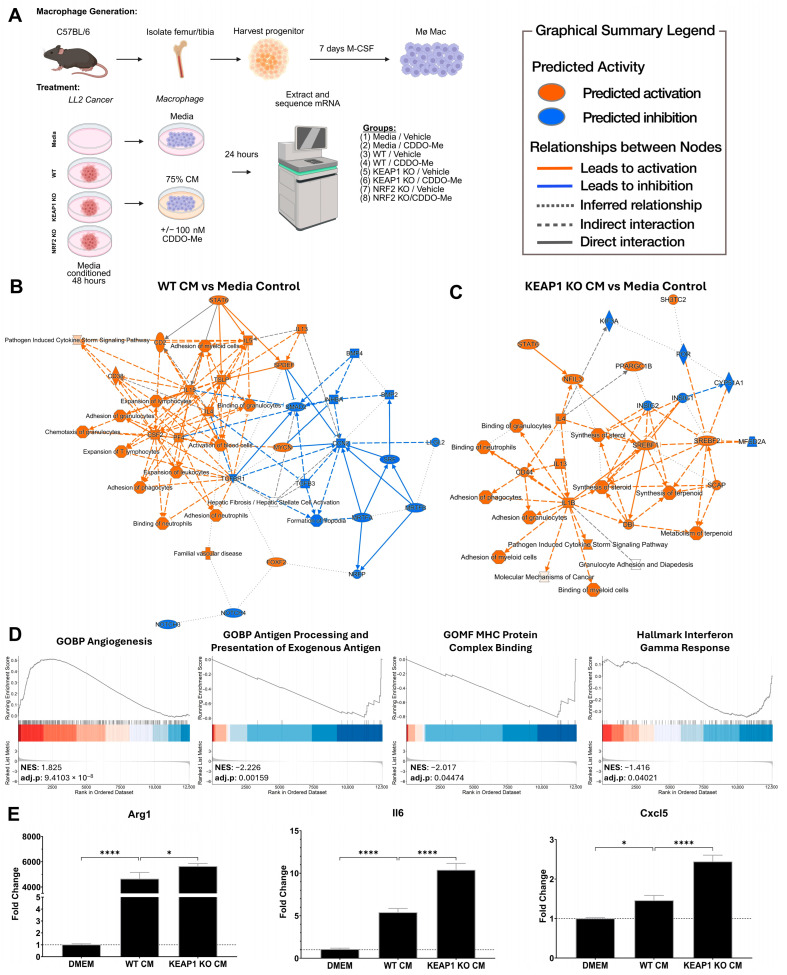
KEAP1 KO lung cancer cells direct pro-tumor polarization of bone marrow-derived macrophages. (**A**) Overall study design for macrophage isolation, differentiation, and treatment. (**B**,**C**) Qiagen IPA graphical summaries of selected bone marrow-derived macrophage (BMDM) RNA sequencing differentially expressed gene contrasts. n = 3 per group. (**B**) LL2 WT cancer conditioned media (CM)-treated BMDM compared against base media control BMDM. (**C**) *KEAP1* KO CM-treated BMDM compared against base media control BMDM. (**D**) Selected gene set enrichment analysis plots for direct comparison of *KEAP1* KO CM-treated BMDM vs. LL2 WT CM-treated BMDM. (**E**) BMDM mRNA expression verification by PCR for macrophage targets and the indicated CM-treatment. Data are mean +/− SE. n = 5. Graphs in (**B**,**C**) are colored by Z-Scores: orange = predicted activation (+) and blue = predicted inhibition (−). Significance determined by one-way ANOVA with Dunnett post hoc test. * = *p* < 0.05 and **** = *p* < 0.0001. (**A**) Created in BioRender. Occhiuto, C. (2025) https://BioRender.com/aka3rm1.

To investigate the regulatory effect of *KEAP1*-KO cancer cells on macrophage polarization in vitro, we used RNA sequencing analysis of primary murine bone marrow-derived macrophages (BMDM) treated with LL2 lung cancer-conditioned media with or without CDDO-Me as shown in Figure 1A. LL2 cells are murine lung carcinoma cells derived from C57BL/6 mice and recapitulate features of human lung adenocarcinoma [48]. *NRF2* KO cancer cells were included as a mechanistic control to eliminate all NRF2-mediated signaling within the cancer cells. All groups were initially compared to the media/vehicle treatment to assess macrophage polarization status, and differentially expressed genes were calculated (Table S1). As compared to media/vehicle BMDMs (naïve state macrophages), WT/vehicle BMDMs had a predicted pathway activation of IL-4 (Z-Score = 5.89), IL-13 (Z-Score = 3.78), and STAT6 (Z-Score = 4.59), which indicate an alternatively activated macrophage (Figure 1B, Table S2). Additionally, there was an increased predicted activation of the downstream functions “Expansion of T lymphocytes” (Z-Score = 2.08), “Expansion of lymphocytes” (Z-Score = 2.23), and “Expansion of leukocytes” (Z-Score = 2.36), which suggests these WT-conditioned macrophages still have the capacity to orchestrate immune responses. For the *KEAP1* KO/vehicle vs. the media/vehicle BMDMs (Figure 1C, Table S2), there was a similar increase in predicted activation of IL-4 (Z-Score = 6.07), IL-13 (Z-Score = 4.55), and STAT6 (Z-Score = 5.17), but the “Expansion of leukocytes” compartments were not enriched. Interestingly, *KEAP1* KO/vehicle BMDMs had an enrichment of lipid-associated pathways such as SREBF1 (Z-Score = 2.10), SREBF2 (Z-Score = 2.59), SCAP (Z-Score = 3.27), and “Synthesis of Sterol” (Z-Score = 2.43). High lipid accumulation in macrophages promotes tumor growth through immunosuppression [49]. It follows that *KEAP1* KO cancer cells induce a metabolic shift in macrophages to promote lipid metabolism, and therefore they decrease anti-tumor immune activating capabilities.

We then assessed specific macrophage functions by computing differentially expressed genes between *KEAP1* KO/vehicle and WT/vehicle BMDMs (Table S3). Using gene set enrichment analysis (Figure 1D, Tables S4 and S5), the *KEAP1* KO/vehicle group had an increased expression of genes involved in angiogenesis (NES: 1.83, *p*.val: 9.41 × 10^−8^) but had decreased antigen processing and presentation (NES: −2.26, *p*.val: 0.0016), MHC binding (NES: −2.02, *p*.val: 0.045), and interferon gamma response (NES: −1.42 *p*.val: 0.040). These findings further indicate a decreased capacity to stimulate an anti-tumor immune response. Finally, we measured specific genes associated with immunosuppressive macrophage phenotype and cancer progression using RT-qPCR (Figure 1E). *Arg1* (*p* = 0.032) [50,51], *Cxcl5* (*p* < 0.0001) [52], and *Il6* (*p* < 0.0001) [53] were all increased in *KEAP1* KO/vehicle-treated macrophages. Taken together, these observations indicate that *KEAP1* KO cancer cells promote a pro-tumor macrophage phenotype at a magnitude greater than the LL2 WT cells.

### 3.2. CDDO-Me Promotes an Anti-Tumor Macrophage Phenotype Regardless of Cancer Cell KEAP1 Status

**Figure 2:**
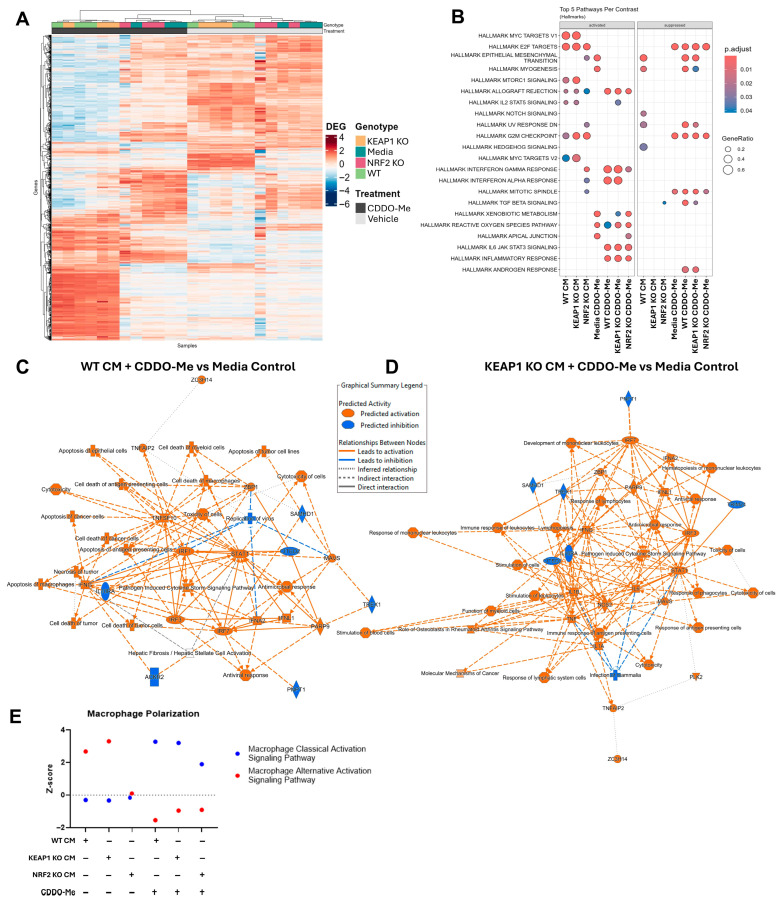
CDDO-Me promotes an anti-tumor macrophage phenotype. (**A**) Heatmap of the top differentially expressed genes in each group meeting a log2-fold change cutoff of +/−1 and *p* < 0.05. (**B**) Top five GSEA enrichments for each group using the mouse “Hallmark” Molecular Signatures Database gene sets. (**C**,**D**) Qiagen IPA graphical summaries of selected BMDM RNA sequencing differentially expressed gene contrasts. (**C**) LL2 WT cancer CM + 100 nM CDDO-Me-treated BMDM compared to media control BMDM. (**D**) *KEAP1* KO CM + 100 nM CDDO-Me-treated BMDM compared to media control BMDM. (**E**) Macrophage polarization status defined by Qiagen IPA canonical pathways for “Classical” and “Alternative” Activation Signaling Pathways. All differentially expressed gene contrasts were compared to media control BMDM.

We next assessed whether CDDO-Me promotes the polarization of anti-tumor macrophages in the context of *KEAP1* KO cancer CM treatment. For each group in the sequencing dataset, differentially expressed genes were calculated as compared to naïve media/vehicle BMDMs. CDDO-Me (100 nM) treatment groups clustered together upon unsupervised hierarchical clustering (Figure 2A). *NRF2* KO CM-treated BMDMs clustered with media-treated macrophages, indicating a higher degree of similarity between these groups, but also that an intact NRF2 pathway in cancer cells is important for promoting the pro-tumor macrophage phenotype. Furthermore, pathways differentially regulated in the *KEAP1* KO and WT CM/CDDO-Me-treated groups align with an anti-tumor macrophage phenotype, evident by the increased interferon gamma (NES: 2.74–2.88, *p* < 1.00 × 10^−9^), interferon alpha (NES: 2.49–2.59, *p* < 1.00 × 10^−9^), and inflammatory responses (NES: 1.93–2.09, *p* = 2.00 × 10^−6^–1.99 × 10^−9^) (Figure 2B, Table S6). Notably, the upregulation of these anti-cancer pathways is independent of the cancer mutation status, as this phenotype was observed in both WT CM/CDDO-Me- and *KEAP1* KO CM/CDDO-Me-treated macrophages.

We then assessed specific regulatory pathways differentially expressed in CDDO-Me treated BMDMs (Figure 2C,D; Table S2). For both WT (Figure 2C) and *KEAP1* KO (Figure 2D) CM-treated BMDMs, CDDO-Me promoted increased signaling in STAT1 (Z-Score = 8.85–8.93), IRF1/3/7 (Z-Score = 7.57–8.74), IFNA1/2/4/17 (Z-Score = 1.91–6.97), and IFNG (Z-Score = 12.11–13.06). As a summary of the overall macrophage phenotype within all treatment groups, we analyzed the Qiagen IPA Canonical Pathways “Macrophage Classical Activation Signaling Pathway” and “Macrophage Alternative Activation Signaling Pathway” for anti-tumor and pro-tumor phenotypes, respectively (Figure 2E, Table S2). The alternative pathway activation was highest in the WT and *KEAP1* CM/vehicle-treated groups (Z-Score = 2.68–3.30), but this phenotype is reversed with CDDO-Me treatment, achieving a phenotype consistent with classical macrophage activation (Z-Score = 3.20–3.28). These data suggest CDDO-Me is an effective regulator of macrophage phenotype in the cancer context, promoting the differentiation of anti-cancer macrophages regardless of the *KEAP1* mutational status of the cancer cell line.

### 3.3. CDDO-Me Slows Tumor Growth in Both WT and KEAP1 KO Flank Tumor Models

**Figure 3:**
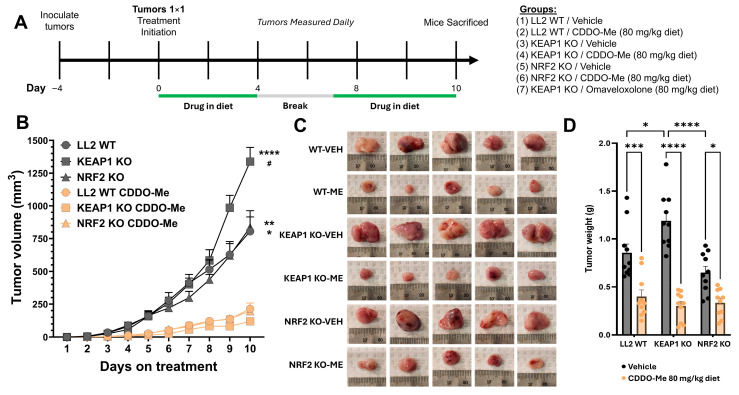
CDDO-Me attenuates tumor growth in both LL2 WT and KEAP1 KO flank tumor models. (**A**) Study design and treatment scheme for C57BL/6 flank tumor model. (**B**) Tumor volume over days of treatment. Tumor volume calculated by ½(*l* × *w* × *h*). *h* = ½(*l* + *w*). (**C**) Representative gross tumor images at takedown. (**D**) Tumor weights for each treatment group immediately after tissue harvesting. Significance determined by two-way ANOVA and Tukey HSD post hoc test. * = *p* < 0.05 and ** = *p* < 0.01. *** = *p* < 0.001 and **** = *p* < 0.0001. For (**B**), * = vs. CDDO-Me for tumor type and # = *p* < 0.05 vs. WT vehicle. Data are mean +/− SE. n = 10 mice per group. VEH = vehicle; ME = CDDO-Me treatment.

Given that CDDO-Me induces an anti-tumor macrophage phenotype regardless of the cancer genotype in vitro, we sought to determine whether *KEAP1* KO tumors respond to CDDO-Me treatment in vivo. We used a flank tumor model in C57BL/6 mice (Figure 3A): the LL2 cell line origin strain. To distinguish the cancer cell-intrinsic effects of triterpenoid treatment from the immune-mediated effects, we included *NRF2* KO cells, which are unable to activate the NRF2 pathway. Mice were inoculated with the indicated cancer cell line and tumors allowed to develop for approximately 4 days. When tumor size reached 1 × 1 mm, mice were randomly assigned to the treatment (80 mg/kg of diet CDDO-Me or omaveloxolone, roughly approximating 20 mg drug/kg body weight) or vehicle diet. A 3-day break from treatment was given to avoid toxicity, and animals were sacrificed 10 days after treatment onset. No behavioral changes were observed, and body weights did not decrease over treatment period, indicating a lack of overt toxicity (Figure S1). *KEAP1* KO tumors had the most aggressive growth kinetics, reaching nearly 1400 mm^3^ after 10 days, whereas WT and *NRF2* KO tumors only reached approximately 800 mm^3^ (Figure 3B,C). *KEAP1* KO vehicle-treated tumors increased in volume and weight by approximately 66% and 39%, respectively (Figure 3B,D), when compared to WT at endpoint. Impressively, the CDDO-Me treatment decreased tumor size across all cancer cell lines (decrease compared to vehicle: WT = 73%, *KEAP1* KO = 91%, *NRF2* KO = 77%), maintaining volumes less than 300 mm^3^ 10 days post-treatment initiation. Tumor weights decreased with CDDO-Me treatment (Figure 3D), regardless of the tumor cell mutational status. These data showcase a compelling treatment effect of CDDO-Me in treating *KEAP1* KO tumors; however, further investigation into the immune microenvironment was required.

### 3.4. The Highly Immunosuppressive KEAP1 KO Microenvironment Reverts with CDDO-Me Treatment

**Figure 4:**
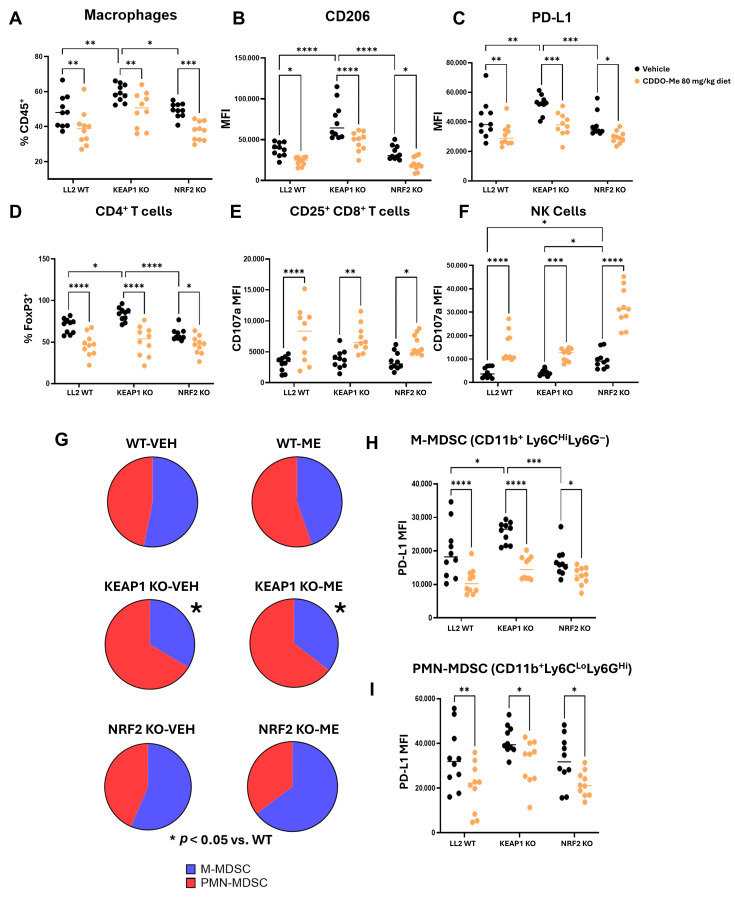
The immunosuppressive KEAP1 KO microenvironment reverts with CDDO-Me treatment. Flow cytometry analysis of the flank tumors in Figure 3. (**A**) Macrophage abundance as a proportion of CD45^+^ cells within flank tumors, and their expression of (**B**) CD206 and (**C**) PD-L1. Lymphoid compartment expression for (**D**) CD4^+^ T cell FoxP3, (**E**) activated CD8^+^ T cell CD107a, and (**F**) NK CD107a. (**G**–**I**) Assessment of myeloid-derived suppressor cells (MDSC). (**G**) Monocytic-MDSC (M-MDSC) and polymorphonuclear-MDSC (PMN-MDSC) as a proportion of total MDSC. PD-L1 expression on (**H**) M-MDSC and (**I**) PMN-MDSC. Significance determined by two-way ANOVA and Tukey HSD post hoc test. (**G**) used a Z-test to determine significance. * = *p* < 0.05, ** = *p* < 0.01. *** = *p* < 0.001, and **** = *p* < 0.0001. Median of the data is indicated by horizontal line. n = 10 mice per group. VEH = vehicle; ME = CDDO-Me treatment.

We evaluated the immune infiltrates in tumors from Figure 3 by flow cytometry (Figure S2) and characterized the immunosuppressive phenotype of *KEAP1* KO tumors. Macrophages increased in *KEAP1* KO tumors by 10.6% of the total CD45^+^ cells (Figure 4A) as compared to WT tumors, with a 92% higher expression of CD206 (Figure 4B) and a 25% increase in PD-L1 (Figure 4C). CD206 is a marker of an immunosuppressive macrophage phenotype and macrophage PD-L1 suppresses the anti-tumor T cell response [54]. Because of the interactions between macrophages and other immune cells, we assessed lymphoid sub-populations, which are a major effector of anti-tumor responses (Figure 4D–F). There was a 13.8% increase in immunosuppressive FoxP3^+^ CD4^+^ T cells as a total percentage of T cells (Figure 4D) in *KEAP1* KO versus WT tumors; however, we did not detect a difference in the activation state of anti-tumor CD8^+^ T cells (Figure 4E) or NK cells (Figure 4F). Myeloid-derived suppressor cells (MDSC) are another important mediator of immunosuppression [55]. We assessed MDSC populations in *KEAP1* KO vs. WT tumors for monocytic-MDSC (M-MDSC: CD11b^+^Ly6C^Hi^Ly6G^−^) and polymorphonuclear-MDSC (PMN-MDSC: CD11b^+^Ly6C^Lo^Ly6G^Hi^) (Figure 4G–I), the two most highly characterized MDSC subtypes. As a proportion of total MDSCs, *KEAP1* KO tumors had an increased proportion of tumor-promoting PMN-MDSC (Figure 4G) compared to WT. This is consistent with the literature that states PMN-MDSCs produce high levels of ROS [56], of which *KEAP1* KO tumors have an increased propensity to detoxify as compared to WT tumors. Immune cells within the TIME likely possess physiological NRF2 activity and are potentially more susceptible to damage from ROS insults generated by PMN-MDSCs. Intriguingly, M-MDSC had increased PD-L1 expression in *KEAP1* KO tumors, despite being the minority fraction of total MDSCs (Figure 4H–I). MDSCs are generally immunosuppressive and tumor-promoting. Their abundance and surface marker expression in the *KEAP1* KO tumor microenvironment may aid in tumor progression, possibly through different mechanisms and subtypes.

Critically, CDDO-Me reverted the immunosuppressive phenotype of the tumor microenvironment in all tumor genotypes, decreasing macrophage infiltration (9.6–12% of CD45^+^ cells; Figure 4A), CD206 expression (34–41% reduction; Figure 4B), and PD-L1 levels (21–26% reduction; Figure 4C). Furthermore, CDDO-Me decreased the percentage of regulatory FoxP3^+^ helper T cells (12–32% decrease in total CD4^+^ T cells; Figure 4D) and promoted the activation of CD8^+^ T cells (Figure 4E) and NK Cells (Figure 4F), as evidenced by the increased expression of the degranulation marker CD107a. While CDDO-Me did not alter the overall proportion of MDSCs (Figure 4G), it did decrease PD-L1 expression on M-MDSC (Figure 4H) and PMN-MDSC (Figure 4I). These findings support the hypothesis that CDDO-Me is an effective immune modulator in cancer and reverts immunosuppressive phenotypes regardless of the cancer *KEAP1* status. The immunomodulatory and tumor-reducing effects of CDDO-Me in the *KEAP1* KO group were of particular interest, as patients harboring these mutations present with more resistant disease and are uniquely difficult to treat. As CDDO-Me is not FDA approved, we therefore worked further to validate the treatment effect (Figure 3) and immune microenvironment changes (Figure 4) in *KEAP1* KO tumors using omaveloxolone.

### 3.5. Omaveloxolone Promotes an Anti-Tumor Microenvironment in KEAP1 KO Flank Tumors

**Figure 5:**
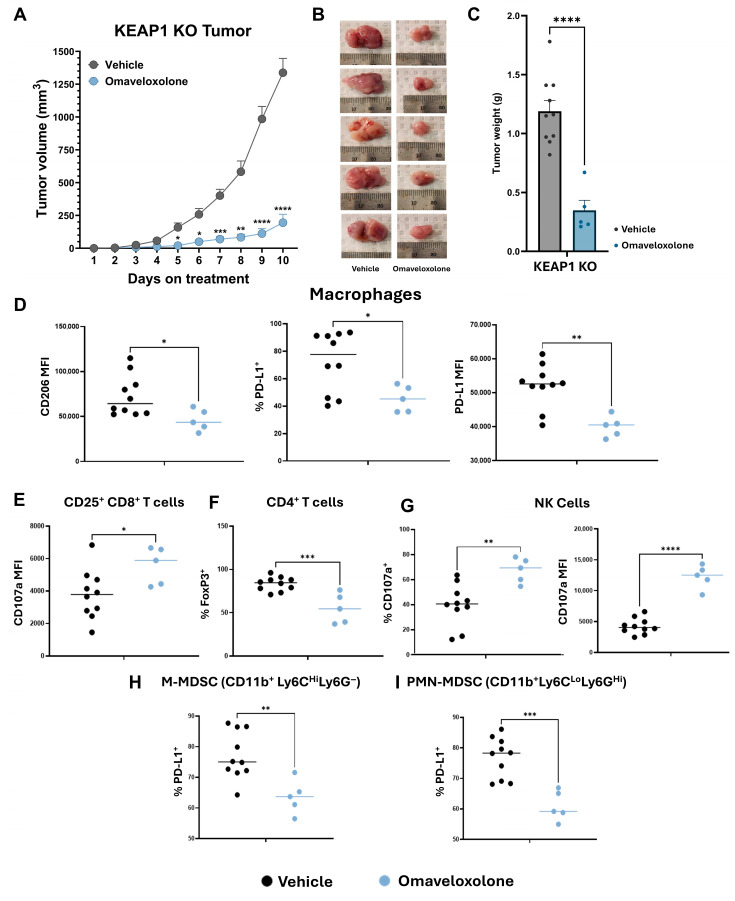
Omaveloxolone promotes an anti-tumor microenvironment in KEAP1 KO flank tumors. Treatment results from the study outlined in Figure 3 for *KEAP1* KO cancer—vehicle vs. 80 mg/kg diet omaveloxolone. (**A**) Tumor growth in *KEAP1* KO flank tumors for duration of treatment. Vehicle cohort is the same as *KEAP1* KO in Figure 3 (**B**) Gross tumor size and morphology (vehicle-treated tumors re-printed from Figure 3) and (**C**) tumor weights at takedown. (**D**) CD206 and PD-L1 macrophage surface markers measured by flow cytometry. Lymphoid compartment surface marker expression for (**E**) activated CD8^+^ T cell CD107a, (**F**) CD4^+^ T cell FoxP3, and (**G**) NK CD107a. PD-L1 expression on (**H**) M-MDSC and (**I**) PMN-MDSC. Significance determined by unpaired *t* test. * = *p* < 0.05, ** = *p* < 0.01. *** = *p* < 0.001, and **** = *p* < 0.0001. Median of data is indicated by horizontal line.

The final arm of the study described in Figure 3 assessed omaveloxolone treatment efficacy and its modulation of the immune microenvironment in *KEAP1* KO vs. vehicle-treated tumors. Omaveloxolone similarly decreased tumor size (Figure 5A,B) and weight (Figure 5C) by 85.4% and 70.7%, respectively, limiting tumor growth to approximately 200 mm^3^ after 10 days of treatment in *KEAP1* KO tumors. Furthermore, the observed changes in the TIME were relatively consistent with the trends observed in *KEAP1* KO tumors treated with CDDO-Me. The expression of the tumor-promoting markers CD206 and PD-L1 were decreased by 36.9% and 23.1%, respectively, on macrophages treated with omaveloxolone compared to the vehicle treatment (Figure 5D). Similarly, activated CD8^+^ T cells displayed increased degranulation, evidenced by the increased expression of CD107a (Figure 5E, 46.9% increase in MFI). Additionally, treatment with omaveloxolone decreased immunosuppressive FoxP3^+^ CD4^+^ T cells by 28.4% (Figure 5F). Finally, there was an increased expression of CD107a on NK cells (Figure 5G, 188% increase in MFI), and a decreased percentage of PD-L1^+^ M-MDSCs (Figure 5H) and PMN-MDSCs (Figure 5I). Taken together, these findings are evidence of a shift in the *KEAP1* KO TIME, from immunosuppressive to anti-tumor, an effect common between omaveloxolone and CDDO-Me. Importantly, these observations paired with the recent FDA approval of omaveloxolone may make this a viable option for treating this subset of cancer.

## 4. Discussion

*KEAP1*-mutant lung cancers represent a highly treatment-resistant patient subset, underscoring the need for further study to better understand and target these tumors. To determine whether NRF2-activated *KEAP1*-mutant cancer responds to triterpenoid therapies, we employed a murine flank tumor model of CRISPR-edited lung cancer. Here, we showed the efficacy of CDDO-Me for effectively treating WT, *KEAP1*, and *NRF2*-mutant murine LL2 flank tumors. We further show that omaveloxolone similarly slows tumor growth in *KEAP1*-mutant tumors. The investigation of the immune microenvironment unveiled that tumors with *KEAP1* mutations promote an immunosuppressive macrophage phenotype both in vitro and in vivo, but triterpenoid treatment promoted anti-tumor macrophage phenotypes in vitro and decreased the expression of immunosuppressive markers in vivo, which likely contributed to the overall decrease in the tumor burden.

Macrophages are orchestrators of immune responses throughout both the innate and adaptive arms of the immune system, and can promote immunosuppression [57]. In assessing the cancer-mediated regulation of macrophages, we found *KEAP1* KO LL2 cancer cells promoted the differentiation of the M2-like alternatively activated phenotype at the RNA level. While both WT and *KEAP1* KO CM induced M2-like macrophages, a distinct phenotype was noted in the *KEAP1* KO CM-treated BMDMs. There were increased lipid-associated pathways, such as *SREBF1/2* (synonymous with *SREBP1/2*). Importantly, lipid metabolism in macrophages can have a profound effect on the microenvironment. Liu et al. noted that fatty acid deficiency within a cancer context leads to mitochondrial damage in macrophages and the subsequent upregulation of *SREBP1*, a regulator of de novo fatty acid synthesis. This dysregulation was associated with the differentiation of M2-like macrophages and the interplay with T cells in the tumor microenvironment which promoted immune evasion [58]. Bidault et al. also found that *SREBP1* depletes macrophages of their antioxidant capabilities, which further promotes M2-like polarization [59]. This decreased antioxidant capacity also suggests that activating NRF2 in these cells would be beneficial. In conjunction with our observed increase in macrophage *SREBP1*, there were simultaneous increases in *Arg1* [50,51], *Cxcl5* [52], and *Il6* [53], all of which can promote cancer progression in different cancers. Because NRF2 is a master antioxidant and metabolic regulator, it follows that perturbations of cancer cell NRF2 regulation would lead to differential macrophage metabolic phenotypes that ultimately promote pro-tumor macrophages. The specific mechanism by which NRF2-activated cancer cells are inducing SREBP1 is not yet known but is conceivably related to fatty acid dysregulation and defective macrophage antioxidant capability. We also observed increased PD-L1 expression on macrophages within *KEAP1* KO tumors. The expression of PD-L1 on macrophages is an established immunosuppressive mechanism, particularly through the inhibition of CD8^+^ T cell activity [60]. Notably, tumor-associated macrophages with high PD-L1 expression have been linked to poor survival outcomes, especially in lung adenocarcinoma [61].

Importantly, our observation that triterpenoids promoted an M1-like macrophage phenotype was supported by increased interferon response-related genes, including STAT1, IRF’s, and IFNG/IFNA regulators, as well as decreased PD-L1 expression. Macrophages are the epicenter of interferon signaling, as they both produce and respond to interferons [62]. Preserving these functionalities, particularly in a cancer context, is critical for maintaining anti-tumor responses, inflammation, and macrophage functionality [63]. In our dataset, CDDO-Me-treated TE-BMDMs phenotypically resembled “classically activated macrophages”, which correlate with favorable prognosis in non-small cell lung cancer patients [64]. In the *KEAP1* KO/CDDO-Me macrophage group, these cells had predicted signaling increases in NOS2 (iNOS), a characteristic trait of M1-like macrophages and a regulator of ROS/redox signaling [65]. Higher IFN, STAT1, and iNOS signaling would indicate the macrophages have a higher capacity for orchestrating anti-tumor immunity [66]. This supports the notion that with CDDO-Me-induced NRF2-activation, which potentially protects against antioxidant- and redox-related damages, macrophages can remain in an M1-like state to facilitate anti-cancer effects. Importantly, this effect is present regardless of the mutational status of the cancer. Given NRF2’s role in regulating oxidative stress and metabolism, its activation may counteract SREBP1-driven lipid accumulation in macrophages, restoring their anti-tumor function.

These immunomodulatory benefits of triterpenoid treatment effect were observed in our animal models as well. Tumor growth was greatly decreased across all three cancer mutation statuses (WT, *KEAP1* KO, and *NRF2* KO). Given the activation of interferon-related pathways in vitro, decreased macrophage CD206 by both triterpenoids (CDDO-Me and omaveloxolone) in vivo further implicates macrophages in the mechanism of triterpenoid anti-tumor efficacy. *KEAP1* KO cancers were previously found to have increased macrophage CD206 [39] and therefore to promote tumor immunosuppression through the generation of M2-like macrophages. Because triterpenoids have a cancer cell mutation-agnostic decrease in macrophage CD206 and increased lymphoid activation, this strengthens support for their potential clinical utility and would indicate *KEAP1*-mutant treatment-resistant cancers may still benefit from triterpenoid therapy. The independence of cancer mutation suggests the great importance of the NRF2 system in immune cells, and its activation outweighs the survival advantage afforded to cancer cells by their NRF2 pathway.

Although macrophages were a central focus of this study, the broader immune microenvironment plays a critical role in shaping tumor progression and therapeutic response. Our flow cytometry analysis revealed that triterpenoid treatment led to the significant restructuring of both lymphoid and myeloid compartments, including increased activation of CD8^+^ T cells and NK cells and reduced frequencies of regulatory T cells. These changes suggest that triterpenoids promote a shift toward an anti-tumor immune landscape. The mechanism underlying this immune remodeling remains unclear, but may involve macrophage-mediated signaling, as our in vitro data indicate that triterpenoids directly influence macrophage polarization. Alternatively, prior work by Torres et al. [67] demonstrated that CDDO-Me can induce the T cell-mediated regulation of macrophages in tri-culture systems, highlighting the complex interplay between immune cell types in response to systemic therapy. In parallel with these immune changes, we observed substantial reductions in tumor size across all genotypes, reinforcing the therapeutic potential of triterpenoids through both immune modulation and tumor-intrinsic effects.

While our study is a proof of concept for treatment efficacy of triterpenoids in a *KEAP1*-mutant cancer setting, some limitations in our model exist. The CRISPR-mediated knockout of *KEAP1* nearly eliminated all *KEAP1* from the cancer cells [39]. This would simulate a cancer cell with maximal NRF2 pathway activity, provided secondary mechanisms of NRF2 inhibition were not activated. In human lung adenocarcinoma, the extent of *KEAP1* inactivation likely varies by patient, with both partial and complete *KEAP1* inactivated mutants existing. The mutant with partial *KEAP1* inactivation may still benefit from further NRF2 pathway activation. Clinically, this may require parsing patient activation states prior to treatment. However, our study in the maximal NRF2 activation state still responds to triterpenoid therapy, even in the *KEAP1*-competent WT cell line. This suggests that the benefit a partial *KEAP1*-mutant cancer may gain from triterpenoid treatment is likely still superseded by the immune-mediated activation within the microenvironment. Furthermore, recent studies have identified BACH1 as a target of CDDO-Me, whose inhibition suppresses cancer cell invasion [68]. These findings indicate a potential tumor cell-intrinsic mechanism that may also contribute to reduced tumor growth. This dual mode of action—immune modulation and BACH1 inhibition—may help explain the broad efficacy of triterpenoids across different *KEAP1* and *NRF2* genotypes.

Future directions for pre-clinical work on this project should involve more mechanistic investigations to delineate the specific cellular contributors to the observed immune modulation. One promising approach would be the use of cell type-specific knockouts of the NRF2 pathway, particularly in macrophages, to establish causality in the context of *KEAP1*-mutant tumors. This strategy would allow for the dissection of NRF2’s role within distinct immune cell populations and help to determine whether its activation is necessary and/or sufficient for the observed anti-tumor effects of triterpenoid treatment. Additionally, in vitro co-culture systems modeling interactions between antigen-presenting cells (e.g., macrophages or dendritic cells) and T cells could provide valuable insight into how NRF2 activation influences adaptive immune responses. These models could be used to assess antigen presentation capacity, T cell activation, and cytokine signaling in the presence of pharmacological or genetic NRF2 modulation. Such studies would be particularly informative in understanding how NRF2-driven metabolic and redox changes in innate immune cells shape downstream T cell responses. Together, these future studies would help clarify the cancer- versus the immune-mediated effects of NRF2 activation and may identify new therapeutic targets or biomarkers for stratifying patients likely to benefit from triterpenoid-based therapies.

Despite efficacy in pre-clinical models, the development of NRF2-activating triterpenoids for cancer treatment has been limited. Two United States-based clinical trials using CDDO-Me for cancer indications have been completed. In a phase I trial assessing CDDO-Me in 44 refractory cancer patients (NCT00529438), two patients had objective responses and ten stable disease [69]. Notably, lung cancer was underrepresented in this study. Due to an incidental finding of improved renal function, clinical development shifted toward chronic kidney disease as the primary indication instead of cancer. Despite this, the authors noted that clinical development in cancer may still be feasible, as even in these refractory patients some stable disease was observed. In a combination phase I/II trial for patients with nonresectable pancreatic adenocarcinoma (NCT00529113), the use of CDDO-Me in combination with gemcitabine therapy promoted immunostimulatory activity [70]. Although, after phase I of this study was completed, it was terminated by the sponsor to pursue other indications. Discontinuation of the clinical development of CDDO-Me has decreased enthusiasm for triterpenoids in cancer treatment, as the clinical course toward therapeutic management of chronic kidney disease was also terminated (NCT03749447). However, in 2023, omaveloxolone was approved by the FDA [30]. After patients experienced significant neurological improvement in the MOXIe trial (NCT02255435) [71], omaveloxolone earned its indication for the clinical management of Friedreich’s Ataxia. With a safer synthetic triterpenoid available for clinical use, it may be time to revisit triterpenoids in cancer therapy.

Our studies here suggest that triterpenoids remain effective for pre-clinical cancer treatment. We have identified a plausible, macrophage-related biological regulation by which the immune microenvironment can be reinvigorated, regardless of the cancer’s *KEAP1* status. In turn, immune stimulation yielded decreased tumor growth. These findings, in combination with safe and viable drug candidates like CDDO-Me or omaveloxolone, warrant further investigation into their clinical utility.

## 5. Conclusions

Taken together, both CDDO-Me and omaveloxolone decreased tumor growth regardless of cancer genotype, and these findings were correlated with beneficial changes to the immune microenvironment. These data support the notion that despite possessing a *KEAP1* mutation, lung cancer may still be effectively treated with an NRF2 pathway activator and the beneficial anti-tumor effects are likely mediated through cancer cell-independent immune regulation.

## Data Availability

The sequencing data generated in this study are available from the Gene Expression Omnibus (GEO), a publicly available data repository. Data are listed under GEO accession GSE308273. All data and project materials used in this study, including cell lines, will be available upon request. Request fulfillment will be made in accordance with the NIH Grant Policy on Sharing of Unique Research Resources (https://grants.nih.gov/grants/policy/nihgps/html5/section_8/8.2.3_sharing_research_resources.htm, accessed on 12 September 2025).

## Supplementary Materials

The following supporting information can be downloaded at: https://www.mdpi.com/article/10.3390/antiox14121406/s1, Table S1: Differentially Expressed Genes vs. Media Control_All Comparisons; Table S2: Qiagen IPA Result Z-Scores vs. Media Control_All Comparisons; Table S3: Differentially Expressed Genes KEAP1 KO vs. WT; Table S4: GSEA Hallmark Pathways KEAP1 KO vs. WT; Table S5: GSEA Ontology Enrichment KEAP1 KO vs. WT; Table S6: GSEA Hallmark Pathways vs. Media Control_All Comparisons; Figure S1: Mouse Body Weights; Figure S2: Overall Immune Cell Abundances.

## Abbreviations

The following abbreviations are used in this manuscript:

TIME	Tumor immune microenvironment
KEAP1	Kelch-like ECH-associated protein 1
NRF2	Nuclear factor erythroid 2-related factor 2
ROS	Reactive oxygen species
LL2	Lewis lung carcinoma
WT	Wild type
KO	Knockout
BMDM	Bone marrow-derived macrophage
M-CSF	Macrophage colony-stimulating factor
SREBP	Sterol Regulatory Element-Binding Protein
CM	Cancer conditioned media
PMN-MDSC	Polymorphonuclear myeloid-derived suppressor cell
M-MDSC	Monocytic myeloid-derived suppressor cell
VEH	Vehicle treatment group
ME	CDDO-Me treatment group
