# Supplementary material for: Synthetic Oleanane Triterpenoids Reduce Tumor Growth and Promote an Anti-Tumor Immune Response Independent of Cancer KEAP1 Mutational Status
Source: Antioxidants (Basel). 2025 Nov 26;14(12):1406. doi: 10.3390/antiox14121406 (PMC12729418; doi:10.3390/antiox14121406)
Supplement: Supplementary file 1 [file antioxidants-14-01406-s001.zip › Figure S2 Overall Immune Cell Abundances.pptx]

## Slide 1
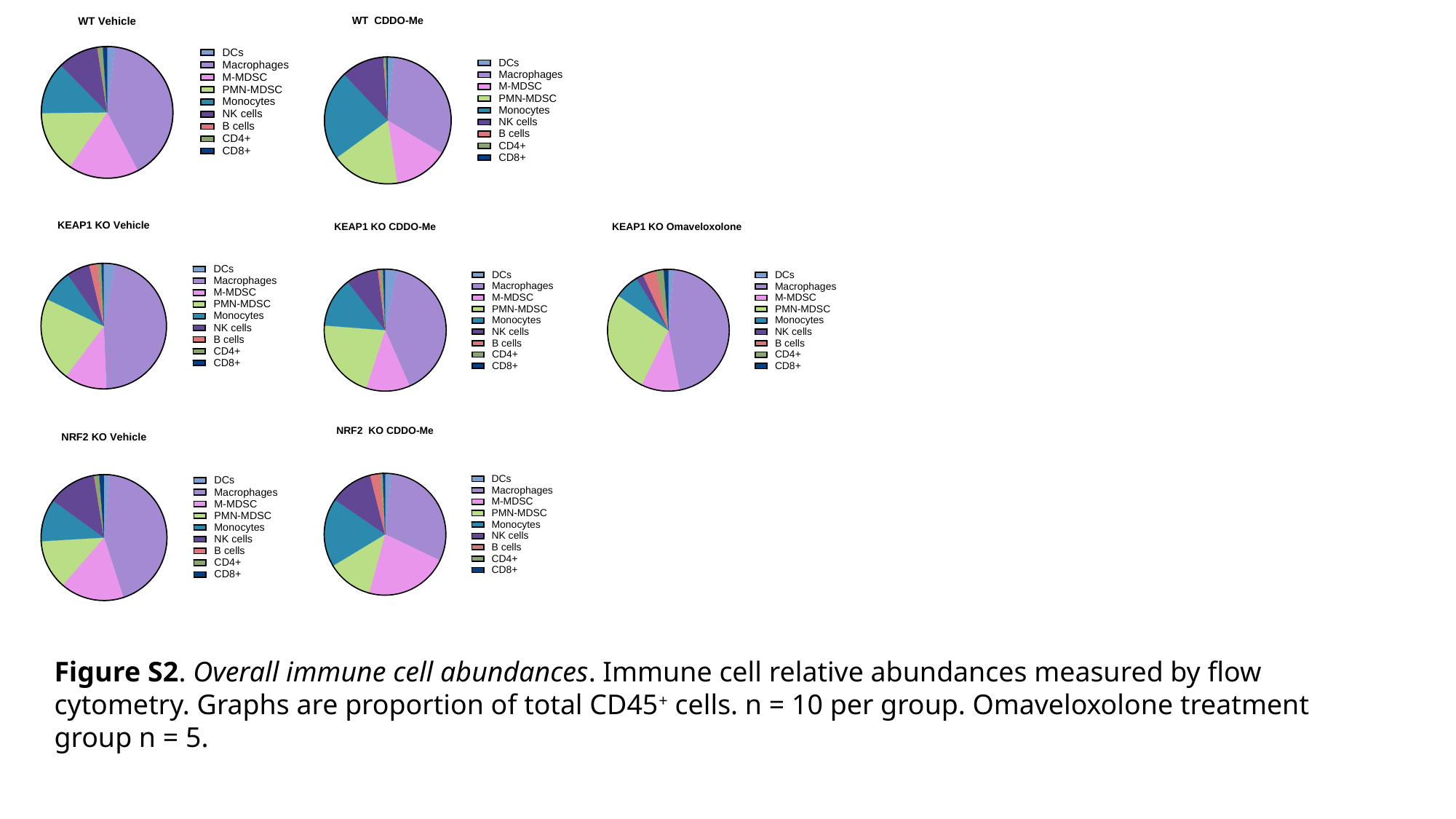

Figure S2. Overall immune cell abundances. Immune cell relative abundances measured by flow cytometry. Graphs are proportion of total CD45+ cells. n = 10 per group. Omaveloxolone treatment group n = 5.
