# Supplementary figures and images for: Synthetic Oleanane Triterpenoids Reduce Tumor Growth and Promote an Anti-Tumor Immune Response Independent of Cancer KEAP1 Mutational Status
Source: Antioxidants (Basel). 2025 Nov 26;14(12):1406. doi: 10.3390/antiox14121406 (PMC12729418; doi:10.3390/antiox14121406)

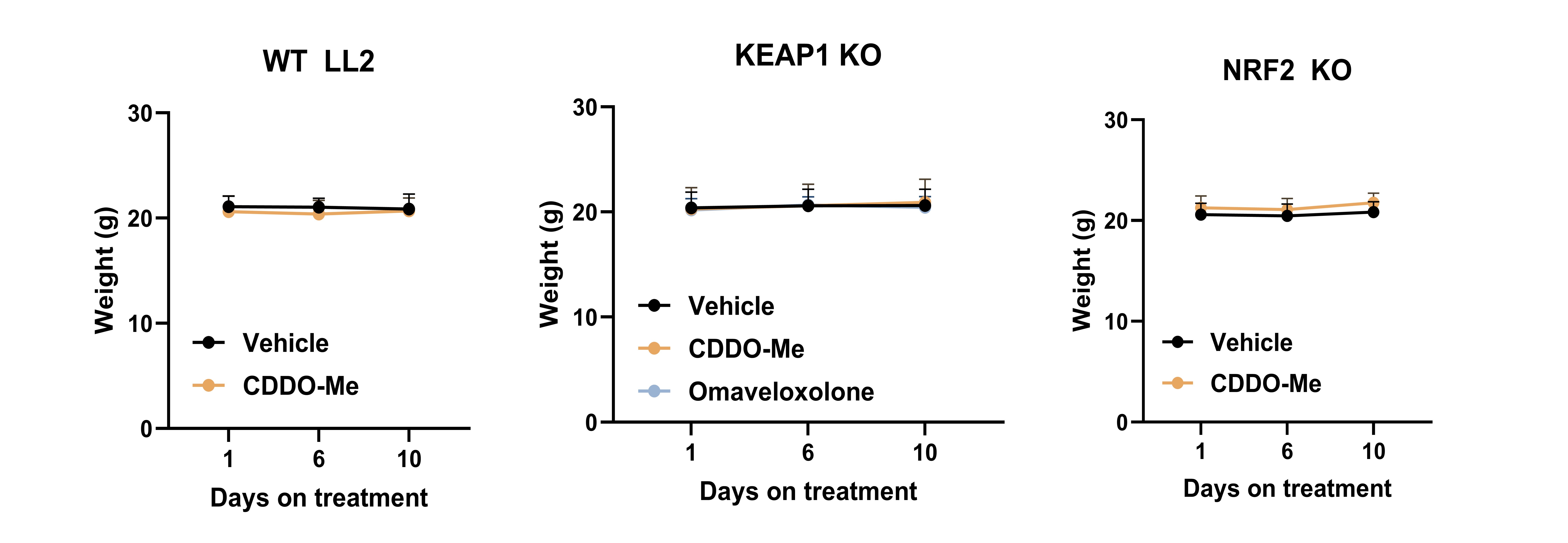

Supplement: Supplementary file 1 [file antioxidants-14-01406-s001.zip › Figure S1 Mouse Body Weights.jpg]

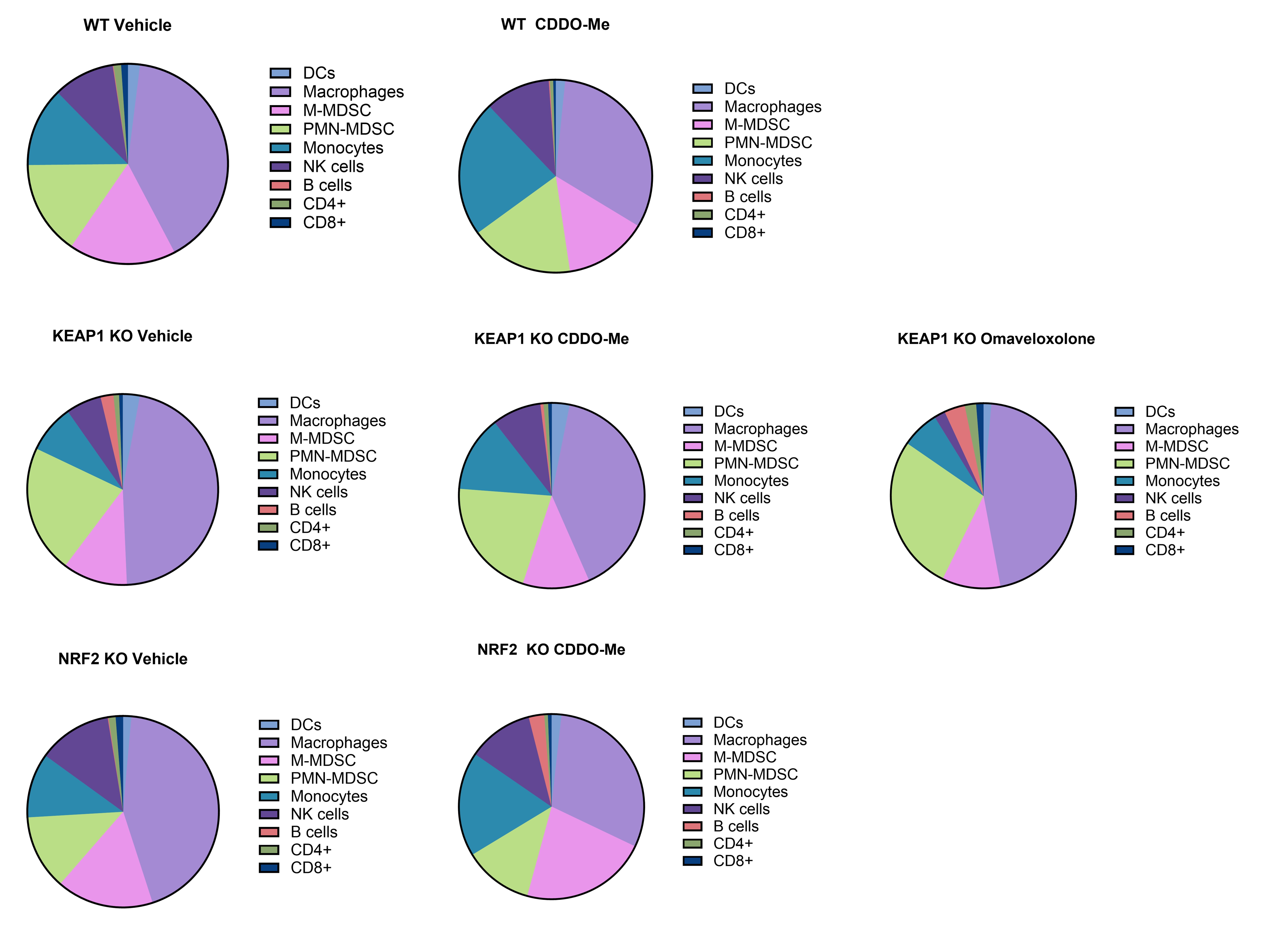

Supplement: Supplementary file 1 [file antioxidants-14-01406-s001.zip › Figure S2 Overall Immune Cell Abundances.jpg]
